# The role of TBK1 in cancer pathogenesis and anticancer immunity

**DOI:** 10.1186/s13046-022-02352-y

**Published:** 2022-04-09

**Authors:** Austin P. Runde, Ryan Mack, Peter Breslin S.J., Jiwang Zhang

**Affiliations:** 1 Department of Cancer Biology, Oncology Institute, Cardinal Bernardin Cancer Center, Loyola University Medical Center, Maywood, IL 60153 USA; 2 Departments of Molecular/Cellular Physiology and Biology, Loyola University Medical Center and Loyola University Chicago, Chicago, IL 60660 USA; 3 Departments of Pathology and Radiation Oncology, Loyola University Medical Center, Maywood, IL 60153 USA

**Keywords:** Autophagy, Cancer pathogenesis, Immunity, Inflammation, Mitophagy, Oncogenesis, Proliferation, Survival, TBK1, TBK1 inhibitor

## Abstract

The TANK-binding kinase 1 (TBK1) is a serine/threonine kinase belonging to the non-canonical inhibitor of nuclear factor-κB (IκB) kinase (IKK) family. TBK1 can be activated by pathogen-associated molecular patterns (PAMPs), inflammatory cytokines, and oncogenic kinases, including activated K-RAS/N-RAS mutants. TBK1 primarily mediates IRF3/7 activation and NF-κB signaling to regulate inflammatory cytokine production and the activation of innate immunity. TBK1 is also involved in the regulation of several other cellular activities, including autophagy, mitochondrial metabolism, and cellular proliferation. Although *TBK1* mutations have not been reported in human cancers, aberrant TBK1 activation has been implicated in the oncogenesis of several types of cancer, including leukemia and solid tumors with *KRAS*-activating mutations. As such, TBK1 has been proposed to be a feasible target for pharmacological treatment of these types of cancer. Studies suggest that TBK1 inhibition suppresses cancer development not only by directly suppressing the proliferation and survival of cancer cells but also by activating antitumor T-cell immunity. Several small molecule inhibitors of TBK1 have been identified and interrogated. However, to this point, only momelotinib (MMB)/CYT387 has been evaluated as a cancer therapy in clinical trials, while amlexanox (AMX) has been evaluated clinically for treatment of type II diabetes, nonalcoholic fatty liver disease, and obesity. In this review, we summarize advances in research into TBK1 signaling pathways and regulation, as well as recent studies on TBK1 in cancer pathogenesis. We also discuss the potential molecular mechanisms of targeting TBK1 for cancer treatment. We hope that our effort can help to stimulate the development of novel strategies for targeting TBK1 signaling in future approaches to cancer therapy.

## Key points

1. TBK1 is activated by PAMPs, inflammatory cytokines and oncogenic kinases.
2. TBK1 coordinates inflammation and metabolism by regulating downstream signaling pathways.
3. TBK1 regulates the proliferation and survival of malignant cells in many types of cancer.
4. TBK1 regulates antitumor immunity and inflammation by regulating cytokine production in dendritic cells and macrophages.
5. TBK1 is a potential molecular anticancer target.

## Background

The TANK-binding kinase 1 (TBK1; also known as NF-κB-activating kinase/NAK and T2K) is a serine/threonine kinase which serves important roles in the regulation of many cellular processes, including innate immunity, inflammatory cytokine production, autophagy, mitochondrial metabolism, and cell survival/proliferation [1–11]. TBK1 can be activated by pathogen-associated molecular patterns (PAMPs; molecules displayed or released by invading bacteria/viruses), damage-associated molecular patterns (DAMPs; molecules displayed or released by damaged tissues), inflammatory cytokines, and oncogenic kinases (in the context of this review, “activated” regarding TBK1 indicates the induction of TBK1 kinase activity by a stimulus; “activated” regarding K-RAS/*KRAS* and N-RAS/*NRAS* specifically indicates the oncogenic activity of these mutated GTPases/proto-oncogenes) [12–18]. The biological activity of TBK1 was first recognized in innate defenses against pathogens for its role in regulating the production of Type I interferons (IFN), including IFN-α and IFN-β. Recent studies have demonstrated that TBK1 links the pathogen-stimulated processes of inflammation/immunity, metabolism, and proliferation involved in many human diseases, including inflammatory diseases, type II diabetes (T2D), obesity, neurodegenerative diseases, and some cancers [19–29]. Mutation-associated haploinsufficiency of the *TBK1* gene has been implicated as a causal event in several types of inflammatory/neurodegenerative diseases, including amyotrophic lateral sclerosis, frontotemporal dementia, Alzheimer’s disease/tauopathies, childhood herpes simplex virus-1 encephalitis (HSE), progressive supranuclear palsy-like syndrome, and a singular case of Parkinsonian-pyramidal syndrome [20, 22, 27, 30–35]. Copy-number gains of the *TBK1* gene have been associated with normal-tension glaucoma and a single case of exfoliation glaucoma, but TBK1 does not appear to be involved in the pathogenesis of high-tension glaucoma; available data additionally suggest TBK1 is not involved in the pathogenesis of juvenile-onset open-angle glaucoma, pigmentary glaucoma, nor steroid-induced glaucoma, but further studies are necessary to confirm this [36–38]. The role of TBK1 in the pathogenesis of inflammatory diseases, T2D, obesity, and neurodegenerative diseases has been discussed in several outstanding reports [1, 21, 26, 39, 40]. In this review, we focus on the role of TBK1 in cancer development, progression, and metastasis in both preclinical animal models and clinical studies using patient samples. We discuss the molecular mechanisms by which TBK1 is regulated in cancer cells and, specifically, the role of TBK1 in the proliferation, survival, and immune system evasion of cancer cells. We expect that this review will provide insightful information and rationales for targeting TBK1 in therapeutic approaches in the field of oncology.

### TBK1 protein structure, interacting partners, and post-translational modification

#### TBK1 protein structure

**Fig. 1:**
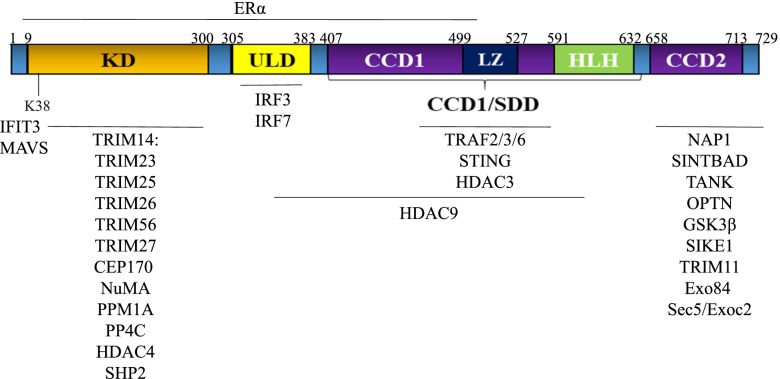
TBK1 protein structure and interaction partners. TBK1 has four archetypical domains: an N-terminal kinase domain (KD), an ubiquitin-like domain (ULD), and two coiled-coil domains (CCD1 and CCD2). The CCD1 domain is also referred to as a scaffold dimerization domain (SDD), which harbors a leucine zipper (LZ) and a helix-loop-helix domain (HLH, aa591-632). The interacting partner proteins of TBK1 are listed and their corresponding binding sites are depicted

The TBK1 protein consists of 729 amino acids (aa). It is conserved across eukaryotes, with human paralogs having been identified in zebrafish, mice, primates, and amphibians. Mouse and human TBK1 share at least 94% sequence homology [41]. TBK1 belongs to the non-canonical IKK family and displays 64% homology to IkB kinase ε (IKKε; also known as IKKi) at the amino acid level. It has four archetypal domains: an N-terminal kinase domain (KD; aa1-307), a ubiquitin-like domain (ULD; aa308-384), and two coiled-coil domains (CCD1; aa407-657 and CCD2; aa659-713) (Fig. 1). The kinase domain is critical for the phosphorylation of various substrates, including IRF3. The Lys38 and Asp135 residues within the kinase domain mediate binding to ATP and catalytic activity, respectively. Both Lys38 and Asp135, as well as Ser172 (a key phosphorylation site), are necessary for the kinase activity and functioning of TBK1 [20, 42–44]. The ULD domain regulates kinase activity by binding to the kinase domain via its hydrophobic patch at Leu352/Ile353 [45]. The ULD also interacts with TBK1 substrates, such as IRF3/7 [45]. TBK1 with ULD deleted or an L352A/I353A mutation is able to induce neither the transcription factors for IFN-β and RANTES (Regulated on Activation, Normal T cell Expressed and Secreted; CCL5) nor phosphorylation of IκBα [46]. The CCD1 is also called the scaffold dimerization domain (SDD), which harbors a leucine zipper domain (LZ; aa499-527) and a helix-loop-helix domain (HLH; aa591-632), both of which mediate dimerization [20]. The CCD2 at the C-terminus harbors an adaptor-binding motif which facilitates the interaction of TBK1 with adaptor proteins, such as TANK, NAK–associated protein (NAP1), TBKBP1 (TBK1-binding protein 1; also known as SINTBAD), or optineurin (OPTN) [20, 43, 47, 48]. These adaptors bind to TBK1 in a mutually exclusive manner, thereby determining the ensuing subcellular localization of TBK1 and consequent downstream signaling specificity [49].

#### Interacting partners of TBK1

Over 50 proteins have been identified as interacting with TBK1 thus far. These interacting proteins can be divided into 3 groups, based on their functions: 1) adaptor proteins for bridging TBK1 to upstream and/or downstream signaling complexes, including TRIF, MyD88, MAVs, TRAF3, TRAF5, TRAF6, TRAF2, TAPE, NEMO, TANK, TBKBP1/SINTBAD, NAP1, STING, IFIT3, RAB8b, DNA-PK, NDP52 and OPTN [44, 47, 49–57]; 2) post-translational regulators of TBK1, including ubiquitin (Ub) ligases (MIB1, NRDP1, UbcH5c, RNF128, DTX4, YAP, TRIP, and members of the TRIM E3 ligase family), the EGLN1 prolyl hydroxylase, deubiquitinating enzymes (DUBs) (A20, TAX1BP1, USP2b, USP38, and CYLD), kinases (SRC, IKKβ, ULK1, GSK3β, DYRK2 and PCKθ), phosphatases (PPM1B, PP4C, Cdc25A, SHP2 and SHIP) and histone deacetylases (HDACs) [58–67]; and 3) substrates that can be phosphorylated by TBK1 and/or mediate TBK1 activity, including molecules involved in IRFs-IFN signaling (IRF3, IRF7, DDX3X, STAT3, STAT6, STING), NF-κB signaling (IKKα, IKKβ, NIK, RelA, cRel, IkBα, NFATc1, TANK, PELI1, ACT1, XIAP, and RIPK1), autophagy (OPTN, p62, CYLD, AMPKα1, RAB7, mLRRK2), proliferation (AKT, mTor, CEP170, NuMA, PLK, and Sec5), insulin signaling (IR; insulin receptor), and neuronal cell functioning (e.g. Ser214 on tau) [10, 15, 24, 27, 39, 42, 54, 57, 68–82] (Fig. 1). The binding sites of many of these partner proteins have been determined (Fig. 1). Generally, adaptor proteins bind to TBK1 at its CCD2 site, post-translational regulators bind to either the KD or CCD1/SDD, and downstream substrates bind to the ULD [45, 49, 83]. Future studies are needed to determine whether these binding partners directly bind to TBK1 or do so indirectly by binding polyubiquitin (poly-Ub) chains or other adaptor proteins.

Each unique upstream stimulus can induce the interaction between TBK1 and a stimulus-specific adaptor protein; as such, the subcellular localization of TBK1 is dependent on and differs according to each stimulus. For example, TANK is localized to the perinuclear region in a punctate-appearing pattern; the binding of TANK to TBK1 induces TBK1-IRF3 pathway activation and the production of IFNα and IFNβ. In contrast, TBK1 binds NAP1 or OPTN in autophagosomes, therein regulating autophagy [71]. Stimulator of interferon genes protein (STING) localizes within the endoplasmic reticulum, while TIR-domain-containing adapter-inducing interferon-β (TRIF, also known as TICAM1) and TBK1-associated protein in endolysosomes (TAPE) localize to endosomes; both are able to induce TBK1-IRF3/IRF7 signaling for type I IFN production [52, 53]. Mitochondrial proteins MAVS and IFIT3 (IFN-induced protein with tetratricopeptide repeats 3) [52, 53] and Golgi complex protein OPTN all promote selective autophagy of damaged mitochondria (i.e. mitophagy) [76]. Thus, subcellular localization of TBK1 is regulated by the selective binding of specific adaptor proteins, which regulate its activity and substrate specificity [82].

#### Post-translational modification of TBK1

**Fig. 2:**
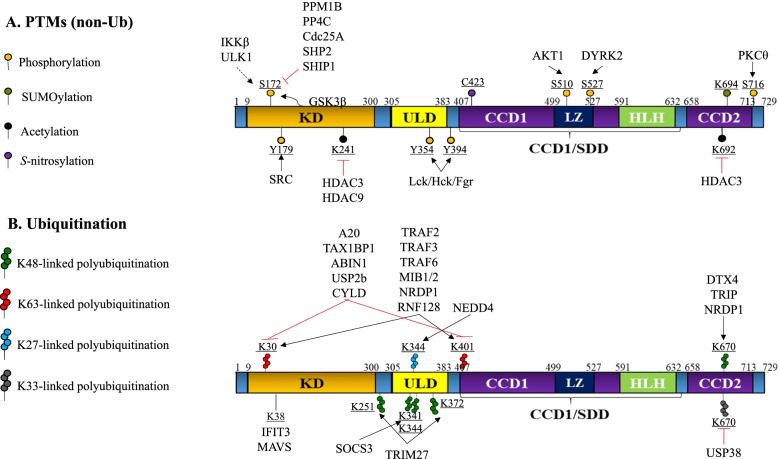
Post-translational modification of TBK1. **A** The proteins that regulate phosphorylation, SUMOylation, and acetylation (non-Ubd PTMs) of TBK1 are listed and corresponding residues are depicted. **B** The proteins that regulate different types of ubiquitination of TBK1 are listed and the corresponding modified residues are depicted

TBK1 protein levels and kinase activity are regulated by post-translational modifications (PTMs), including oligomerization, phosphorylation, ubiquitination (Ubn), acetylation, SUMOylation, and adaptor protein-interaction (Fig. 2).

Inactive TBK1 is present throughout the cytosol as a compact homodimer mediated by ULD and CCD1/SDD interactions [43, 44]. Upstream signaling induces a multistep mechanism of TBK1 activation, including K63-linked polyubiquitination (poly-Ubn) and adaptor-protein interaction, followed by Ser172 phosphorylation [43, 84–89]. K63-linked poly-Ubn of TBK1 on K30 and K401 is mediated by the E3 Ub ligases TRAF2/3/6, MIB1/MIB2, NRDP1, and RNF128 in response to different types of stimuli, including viral RNA/DNA and bacterial LPS [16, 84–86, 90–94]. K30 and K401 poly-Ubn provides a platform for both adaptor protein (e.g. NEMO, TBKBP1/SINTBAD, NAP1, NDP52, and OPTN) and substrate (e.g. IRF3/7) binding, to propagate downstream signaling activity [95]. K63-linked poly-Ubn also promotes the interaction of the CCD2 domain with adaptor proteins. The TBK1 adaptor proteins TANK, NAP1, TBKBP1/SINTBAD, and OPTN all have distinct subcellular localizations. As these proteins complex with TBK1, they ultimately promote the consequent localization and sequestration of TBK1 in their respective cytosolic compartments. As such, TBK1 adaptor proteins can promote the formation of higher-order TBK1 oligomers, therein forming TBK1-adaptor protein signaling machinery in distinct, functionally-specific subcellular locations [43, 44, 83, 96]. This results in regional activation of TBK1 by trans-autophosphorylation on Ser172 within the kinase activation loop (aa160–175), an essential step for kinase activation [43, 44, 97, 98].

TBK1 has also been observed to undergo SUMOylation on its C-terminal K694 [88]. Such SUMOylation sterically excludes some adaptor proteins, like TANK, allowing TBK1 to interact with other protein complexes, thereby enabling its innate immunity-activating function.

Although Ser172 phosphorylation of TBK1 is primarily mediated by autophosphorylation, it can also be phosphorylated by IKKβ, suggesting crosstalk with the IKK protein family, and by ULK1 as part of the inflammatory response to antigen detection [39, 99]. In addition, autophosphorylation of Ser172 is regulated by phosphorylation of other residues by several kinases. For example, after being recruited to the TNF-receptor associated factor 3 (TRAF3) complex, GSK3β complexes with TBK1 to mediate activation of TBK1 via autophosphorylation on Ser172 independent of GSK3β kinase activity [75, 97]. Furthermore, in K-RAS-activated lung epithelial cells, PKCθ is activated by mutant K-RAS, which phosphorylates TBK1 on Ser710 in a TBKBP1- and CARD10-dependent manner, triggering the autophosphorylation and activation of TBK1 [100]. Src binds to adaptor proteins (such as TRIF, MAVS, and STING) and phosphorylates TBK1 on Tyr179, which is crucial for the TBK1-mediated activation of IRF 3[101]. During RNA virus infections, MAVS-mediated TBK1/IKKε activation requires both TRAF-mediated TBK1 autophosphorylation and TRAFs-NEMO-IKKβ-mediated TBK1 phosphorylation [86].

Since TBK1 activation is primarily mediated by autophosphorylation and K63-linked poly-Ubn, aberrant TBK1 activation is prevented by both phosphatases and DUBs. Several phosphatases, including PPM1B, PPM1A, PP4C, Cdc25A, SHP2, and SHIP1, have been reported to suppress TBK1 activity via phosphatase action on Ser172 of TBK1 [43, 62–64, 66, 97, 98, 102]. In addition, several DUBs, including A20, TAX1BP1, USP2b, and CYLD, have been shown to remove K63-linked poly-Ub chains from TBK1, thereby inhibiting the TBK1-IRF3 signaling pathway [61, 103–105]. Moreover, TBK1 can also be negatively regulated via phosphorylation. For example, Lck/Hck/Fgr-mediated phosphorylation on Y394/354 of TBK1 disrupts its dimerization, and thus its activation, during the innate antiviral response [106]. DYRK2-mediated phosphorylation of Ser527 triggers DTX4 or TRAIP-mediated K48-linked poly-Ub on K670, leading to proteasomal degradation [91, 107–110]. Interestingly, this K670 residue can be modified through K33-linked poly-Ub by a currently unidentified Ub ligase [108]. USP38 specifically cleaves K33-linked poly-Ub chains from TBK1 at K670, thereby enabling subsequent K48-linked Ubn by DTX4 and TRIP on the K670 residue. USP38 also inhibits type I IFN signaling by promoting the NLRP4 signalosome-mediated degradation of TBK1 [108]. In addition, the interaction of TRAF3IP3 with TRAF3 and TBK1 induces DTX4-mediated K48-linked Ubn of TBK1 on its K372 residue, therein promoting degradation of TBK1 [107]. As well, NEDD4 catalyzes the K27-linked poly-Ubn of TBK1 on its K344 residue, which serves as a recognition signal for cargo receptor NDP52-mediated autophagic degradation [8]. Siglec1 suppresses the antiviral innate immune response by inducing TBK1 degradation, wherein Siglec1 induces the TRIM27 Ub ligase to mediate K48-linked Ubn of TBK1 on K251 and K372 [111]. In response to infection with an RNA virus, TBK1 is ubiquitinated (Ubd) on residues K69, K154, and K372; residues K69 and K154 are critical for innate antiviral responses and IFN production [112]. TRAF-interacting protein (TRIP) promotes K48-linked Ubn and consequent proteasomal degradation of TBK1, thus inhibiting TLR3/4- and RIG-I-induced IFN-β signaling [110]. SOCS3 catalyzes K48-linked poly-Ubn of TBK1 on residues K341 and K344 and promotes subsequent TBK1 degradation [113]. Furthermore, YAP/TAZ interacts with TBK1 and blocks MIB2-mediated K63-linked poly-Ubn of TBK1 and its adaptor proteins, thus inhibiting the association of TBK1 with IRF3, MAVS, and STING [114–116].

TBK1 activity is also positively and negatively regulated by acetylation [59]. Nine lysine residues on TBK1 can be modified by acetylation: K30, K154, K236, K241, K251, K607, K646, K691, and K692, as indicated by mass spectrometry. K241 acetylation during the early stage of viral infection enhances the recruitment of IRF3 to TBK1. Deacetylation of K241 and K692 is critical for the kinase activity and dimerization of TBK1, respectively [59]. The acetyltransferases which mediate TBK1 acetylation are not yet known. However, several HDACs have been reported to either positively or negatively regulate TLR-mediated and virus-stimulated innate immune responses. HDAC3 directly deacetylates TBK1 on K241 and K692, resulting in the activation of TBK1. Interestingly, HDAC3 activity is promoted when TBK1 is phosphorylated on Ser424. HDAC9 deacetylates TBK1 on K241 for activation of antiviral innate immunity, which is enhanced by the methyltransferase DNMT3A [58]. HDAC4 interacts with the kinase domain of TBK1/IKKε, blocking the phosphorylation of IRF3 in order to suppress IRF3-mediated IFNβ expression [117].

A recent study demonstrated that TBK1 undergoes a downregulatory *S*-nitrosylation on its Cys423 residue [118]. The study suggested that Cys423 *S*-nitrosylation occurs due to reactive nitrogen species produced during viral infection. As such, it is suggested that GSNOR prevents *S*-nitrosylation of TBK1, thereby preserving the immune-activating functions of TBK1 amid viral infection.

TBK1 levels are also regulated by autophagy, such that inhibition of autophagy can result in aberrant activation of TBK1, suggesting that the activity of TBK1 is subject to negative regulation by autophagy, at least in K-RAS-activated murine pancreatic cells [9].

### TBK1-mediated signaling pathways

#### Upstream signaling stimulates TBK1 activation

**Fig. 3:**
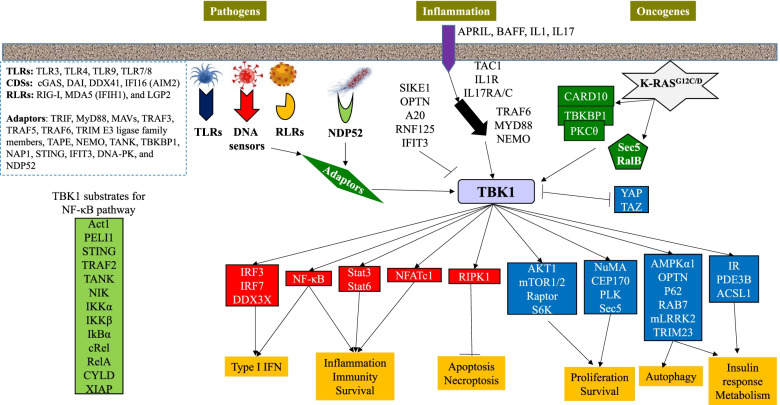
TBK1-mediated signaling pathways. TBK1 activation is stimulated by pathogenic infections and inflammatory cytokines via corresponding receptors bridged by adaptor partners. In addition, activated K-RAS also activates TBK1 in tumor cells. Active TBK1 promotes anti-pathogen immunity and inflammatory cytokine production by stimulating IRF3/7-IFN, NF-κB, NFATc1 and STAT3/6 pathways, cellular proliferation and survival by inducing the AKT-mTOR and PLK1/CEP170 mitotic pathways, as well as mitochondrial and insulin metabolism by inducing autophagy and insulin receptor signaling

TBK1 can be activated by viral and bacterial invasion, inflammatory cytokines, and oncoproteins (such as activated K-RAS) [119]. During pathogen infection, pattern-recognition receptors (PRR) allow for the cellular recognition of conserved molecular signatures of microbial PAMPs, which activate TBK1. Based on protein domain homology, PRRs have been divided into several families, including: Toll-like receptors (TLRs), retinoic acid-inducible gene I (RIG-I)-like receptors (RLRs), cytosolic DNA sensors (CDS), NOD-like receptors (NLRs), and absent in melanoma-2 (AIM2)-like receptors (ALRs) [12–14] Fig. 3.

Human TLRs consist of 10 family members which can be divided into 2 subgroups, cell surface TLRs (including TLR1, TLR2, TLR4, TLR5, TLR6, and TLR10) and endosomal TLRs (including TLR3, TLR7, TLR8, and TLR9) [120]. Different TLRs recognize distinct types of PAMPs; cell surface TLRs mainly recognize the membrane components of pathogens, while endosomal TLRs primarily recognize cytosolic nucleic acids. For example, TLR2 along with either TLR1 or TLR6 recognize lipoproteins, peptidoglycans, lipotechoic acids, zymosan, mannan, and tGPI-mucin; TLR3 detects viral dsRNA and poly(IC); TLR4 recognizes bacterial lipopolysaccharide (LPS); TLR5 recognizes bacterial flagellin; TLR7/8 detect ssRNA; TLR9 detects unmethylated CpG DNA molecules; and TLR10 senses influenza A virus (IAV) infection and cooperates with TLR2 to recognize the PAMPs of *Listeria*. After binding to their corresponding PAMP(s), most TLRs stimulate the activation of myeloid differentiation factor 88 (MyD88)-IRAK1/IRAK4- TRAF6, NF-κB, and TBK1 signaling, while only TLR3 and TLR4 stimulate TRIF-TRAF3-TBK1 signaling in endosomes [121].

There exist 3 RLRs: RIG-I, melanoma differentiation-associated protein 5 (MDA5), and laboratory of genetics and physiology 2 (LGP2) [122]. RLRs detect foreign RNA in the cytoplasm. After detecting non-self RNA, RIG-I and MDA5 undergo conformational changes which expose and multimerize their caspase activation and recruitment domains (CARDs), which interact with MAVS on mitochondria via CARD-homotypic interaction, thereby inducing MAVS oligomerization [123–129]. MAVS then recruits the Ub E3 ligases TRAF2, TRAF5, and TRAF6 to synthesize poly-Ub chains which consequently recruit the adaptor proteins TANK, NAP1, or TBKBP1 for TBK1 activation [122]. However, as it lacks a CARD, LGP2 negatively regulates RIG-I-mediated recognition of viral dsRNA, reduces the production of IFNs and inflammatory factors, and ultimately inhibits the antiviral innate immune response [130]. However, LGP2 facilitates the antiviral response mediated by MDA5 [131–133].

The CDS family has multiple members including cGAS (cyclic GMP-AMP (cGAMP) synthase), DNA-dependent activator of IRFs (DAI, as known as ZBP1), interferon gamma inducible protein 16 (IFI16), and DEAD-Box Helicase 41 (DDX41) [134, 135]. CDSs detect foreign double-stranded DNA (dsDNA) in the cytoplasm. Activated CDSs induce STING in the endoplasmic reticulum, which then translocates to the Golgi and recruits TBK1 [76, 83, 87, 136, 137]. Among these 3 CDSs, dsDNA stimulates a conformational change in cGAS that allows ATP and GTP to enter the catalytic pocket, leading to the synthesis of cGAMP, a potent activator of the STING-TBK1 axis. During intracellular bacterial infection, autophagy receptor NDP52 recognizes Ub-coated bacteria and recruits the adaptor proteins NAP1 and TBKBP1 to activate TBK1 [138]. TBK1 then promotes autophagy and the invading microbial pathogens can be selectively eliminated (xenophagy) [4, 5, 7]. Furthermore, TBK1 can also be activated by inflammatory cytokines including APRIL, BAFF, IL-1, and IL-17, as well as by activated K-RAS in cancer cells, as will be discussed in later sections [16–18, 78, 139–141].

#### Downstream substrates and signaling pathways of TBK1

TBK1 is a key mediator of immune response/inflammation, autophagy, proliferation/growth, survival, insulin signaling and metabolism. Mass spectrometric analysis of *TBK1* knockdown in lung cancer cells identified 385 proteins with altered phosphorylation [142]. Over 50 substrates have been identified experimentally thus far. These can be divided into several groups based on their functions and the signaling pathways they impact: immune response/inflammation (IRF3, IRF7, DDX3X, IKKα, IKKβ, NIK, RelA, cRel, IkBα, ACT1, Peli1, STAT1, STAT3, STAT6, XIAP), autophagy (OPTN, LC3C, CYLD, AMPKα1, RAB7, GABARAP-L2, p62/SQSTM1, mLRRK2, Stx17, PINK1/Parkin), cellular proliferation and mitosis (Akt, mTOR, Raptor, mTORC1, P70S6, GSK3α, CEP170, NuMA, PLK, metadherin, Sec5, Cdc20, and Cdh1), insulin and metabolic signaling (insulin receptor, PDE3B and ACSL), and apoptosis/necroptosis (RIPK1) [6, 42, 54, 57, 69, 70, 73, 142–145]. As previously mentioned, several adaptor proteins such as STING, TANK, TRAF2, TRIF, MAVS, and MITA are also substrates for TBK1 (Fig. 3 and Table 1). While the roles of TBK1 in insulin signaling, metabolism, and neurodegenerative disease have been discussed in several excellent reviews [21, 82], we will focus on the roles of TBK1 in the regulation of inflammation/immunity, cellular proliferation/survival, and autophagy due to the involvement of these processes in oncogenesis and disease progression.

**Table 1:** The substrates of TBK1 and their corresponding signaling pathways

Pathways	Substrates
IRF3/7-IFN pathway	IRF3 at Ser339/386/396 [146–151] IRF7 Ser471/472 [152, 153] DDX3X at Thr542, Ser543 [42] STAT3 at Ser754, 727 [72, 154] STAT6 at Ser407, Tyr641 [155]
NF-κB signaling	PEL1 Ser76/293, Thr288 [156] IKKβ at Ser177/181 [41] NIK at Ser862 [78] XIAP at Ser430 [157] CYLD at Ser418 [158] RIPK1 at Thr189 [74] p65 NF-κB (RelA) at Ser536 [70, 159, 160] cRel at Ser479/602 [70] IkBα at Ser36 [41, 161] Metadherin at Ser568 [142] ACT1/TRAF3IP2 at Ser162/220/ 233/311 [162]
Mitotic regulators	PLK1 at Ser568, Thr210 [142] CEP170 at multiple sites [80] NuMA at multiple sites [80] Cdc20 at Ser134 [163] Cdh1 at Thr20, Ser39/42/58 /131/151 [163]
AKT-mTor signaling	AKT1 at Thr195/308, Ser378/ 473/476 [2] Raptor at Ser877 [15] mTor at Ser2159/2481 [68, 164] S6K at T421/S424 [165] Sec5 [69] GSK3α at Ser21 [44] SRC at Tyr179 [101]
Autophagy pathway	AMPKα1 at Ser459/476 [39], Thr172 LC3C at Ser93/96 [71] GABARAP-L2 at Ser87/88 [71] p62/SQSTM1 on Ser403/366 [4] mLRRK2 at Ser910/935/995 [166] OPTN at Ser177/473/513 [54, 167] STX17 at Ser202 [73] RAB7A at Ser72 [54]
Adaptor proteins	STING Ser324/326/358/366 [134, 168, 169] TRIF at Ser210/212, Thr214 [169] MAVS at Ser442 [169] MVB12b at Ser222 [170] TANK Ser49/126/178/208/228/257 [99] TRAF2 at Ser11 [171]
Insulin/glucose signaling	IR at Ser994 [25] PDE3B at Ser 318 [172] Exo84 Ser8 [173]
Others	Tau at Ser214/324/356 [27] HTT at Ser13/16 [28] ERα at Ser-305 [174] YAP1 at Thr110/114, Ser128/131 HDAC3 at Ser424 [59]

#### TBK1 is a critical mediator of immunity and inflammation

Upon pathogen detection, TBK1 is induced by upstream adaptor proteins and receptors. TBK1 is an early effector of the innate immune system, with its activation occurring very shortly after pathogen detection. The primary function of TBK1 is to induce production of type I IFN, such as IFN-α/β, in innate immune cells; TBK1 does so largely via its association with and phosphorylation of IRF3 and IRF7 [175–177]. Activated TBK1 directly phosphorylates IRF3/7 on multiple Ser and Thr residues. Phosphorylation of Ser386 and Ser396 on IRF3 is believed to be critical for IRF3 activation [146–148], while phosphorylation of Ser 477 and 479 of IRF7 is believed to be necessary for IRF7 activation [178]. The phosphorylated IRFs form homo- and/or hetero-dimers, translocate into the nucleus, and bind IFN-stimulated response elements (ISRE) in the promoters of target genes, such as *IFNB* and *RANTES* [134]. IRF dimers subsequently regulate target gene expression by recruiting p300 and CBP co-activators [179], and by cooperating with several other transcription factors, such as NF-κB, PU.1, and SMADs [180]. In addition, the transcriptional activity of IRF3 is regulated by PTEN, by DNA-PK and by DDX3X (a DEAD-box helicase family member). PTEN promotes activation of the innate immune system via phosphatase action on Ser97 of IRF3, an inhibitory residue, thereby enabling the nuclear translocation of IRF3 [181]. DNA-PK phosphorylates IRF3 on Thr135, inducing the nuclear retention of IRF3, thereby extending the half-life and thus the transcriptional activities of IRF3 [182]. TBK1 phosphorylates DDX3X on multiple sites and then the phosphorylated DDX3X is associated with TBK1 to modulate IRF3 activation and IFN-β production [42].

TBK1 activates NF-κB signaling by phosphorylating several members of this pathway, including RelA, cRel, and IkBα [41, 160, 161, 183]. Activation of NF-κB signaling promotes inflammation through the production of inflammatory cytokines, including TNFα, IL-8 and IL-1β, and induces proliferation by regulating survival/proliferative genes, including *BCL-xL*, *XIAP*, *Cyclin D1*, and *RelB* [184]. TBK1-mediated activation of NF-κB seems highly dependent on cell- and signal-specific contexts [119]. TBK1 mediates cytosolic DNA-induced/STING-dependent activation of both the IRF3 and NF-κB pathways [83, 185]. NF-κB cooperates with IRF3 to induce production of proinflammatory cytokines, including type I IFN [186]. The activation of both the IRF3 and NF-κB pathways mediates an immune defense against both tumors and viruses [83, 185]. Understanding these functions of TBK1, we can understand why patients with heterozygous loss-of-function *TBK1* mutations are susceptible to herpes simplex infection, as observed in HSE patients [35].

In addition to the IRF and NF-κB pathways, TBK1 can also activate STAT3, and STAT6, thereby promoting an inflammation-induced innate immune response. In multiple cell lines, TBK1 directly phosphorylates STAT3 on Ser754, thereby restricting cytosolic DNA-induced STAT3 transcriptional activity [72]. In dendritic cells (DCs), TBK1 binds to and phosphorylates STAT3 on Ser720, thereby suppressing both the type I IFN response and STAT1 activation [154]. Regarding STAT6, TBK1 phosphorylates STAT6 on Ser407, leading to the dimerization and nuclear translocation of STAT6, and consequent production of STAT6-responsive genes, including the chemokines CCL2/20/26 [155]. Thus, TBK1 acts as a central player in the innate immune system by regulating inflammation during the response to pathogens.

Interestingly, germline knockout of *Tbk1* in C57BL/6 mice is embryonic-lethal by embryonic day 14.5. This is due to unrestrained apoptosis and necroptosis in murine hepatocytes, resulting in severe inflammation and eventual liver failure [161]. Subsequent investigation determined that the embryonic lethality observed in *Tbk1*^−/−^ C57BL/6 mice was a result of TNFα-stimulated hyperactivation of RIPK1. Mechanistically, TBK1 attenuates the activation of RIPK1 by phosphorylating it on Thr189, thus mediating retention of RIPK1 in the survival complex. In TBK1-deficient cells, merely physiological levels of TNFα can stimulate the release of RIPK1 from the survival complex; as such, the uninhibited RIPK1 subsequently forms a death complex in the cytosol and induces both caspase 8-mediated apoptosis and RIPK3-MLKL-mediated necroptosis [74, 145]. Either TNFα deletion or RIPK1 kinase inactivation can prevent the early embryonic death of *Tbk1*^*−/−*^ C57BL/6 mice. In addition, germline *Tbk1*^−/−^ mice with a 129S5 background are able to survive due to the lack of the *Tnfrsf1b* gene [187]. As cells that die from necroptosis are highly immunogenic/inflammatory, mice with *Tbk1* loss-of-function mutations display pathological, whole-body inflammation compared to wild-type mice [188]. Thus, TBK1 has been shown to play a critical role in the prevention of inflammation by suppressing TNFα-RIPK1-mediated cell death. Consistent with these studies in animal models, recently, homozygous point mutations of TBK1 (including W619X, Y212D, and R440X) were detected in 4 patients with an unidentified chronic, systemic autoimmune syndrome [189]. All these mutations are loss-of-function mutations. The W619X and R440X mutations induce nonsense-mediated mRNA decay and cause markedly reduced *TBK1* mRNA levels, while the Y212D mutation attenuates IRF3-IFN signaling activity. Although *TBK1*-null cells display compromised TLR3-TRIF and cGAS-STING signaling, RLR-MAVS signaling is maintained, suggesting that the loss of TBK1 is partially compensated by IKKε. Consequently, all four patients presented with normal antiviral immune function. The autoimmune symptoms which developed in these patients might be associated with over-activation of TNFα signaling because they could be ameliorated by anti-TNFα therapy [189]. Such a clinical observation is consistent with the laboratory findings in animal models that TBK1 restrains TNF-induced RIPK1-MLKL activation [74, 145].

#### TBK1 is a key regulator of selective autophagy and xenophagy

Autophagy is a homeostatic cellular process by which cellular components, such as organelles or protein aggregates, are degraded in a lysosome-dependent manner [190]. It is an important process by which cellular nutrients are recycled, damaged/unneeded organelles are resolved, and the health of the cell is maintained [190]. Following the inhibition of mTOR or the activation of AMPK, and subsequent formation of the unc-51-like kinase 1 (ULK1) complex (ULK1/2, ATG13, FIP200/RB1CC1 and ATG101), autophagy is initiated via the nucleation of the phagophore upon budding from the surface of the endoplasmic reticulum [21, 191–193]. The ULK1 complex recruits and phosphorylates the transmembrane protein ATG9 and the class III phosphatidylinositol 3-kinase (PI3KC3) complex (PIK3C3/VPS34, PIK3R4/VPS15, beclin-1, ATG14, as well as associated factors AMBRA1 and NRBF2), which in turn promotes autophagosome biogenesis [190]. The ATG16L1 complex of lipidation cascade enzymes (ATG3, ATG7, ATG12-ATG5-ATG16L1) induces the conjugation of Atg8/LC3/GABARAPs (autophagy modifiers) to phosphatidylethanolamine by facilitating ATG4-mediated lipidation of LC3-I to form LC3-II and anchor LC3-II to the growing phagophore. LC3-II supports both expansion and closure of the autophagosome (double-membraned vesicle), allowing it to properly engulf its targets. Autophagosomes are studded with RAB7, which facilitates fusion with lysosomes to form autolysosomes for the degradation of the substrate/cargo.

**Fig. 4:**
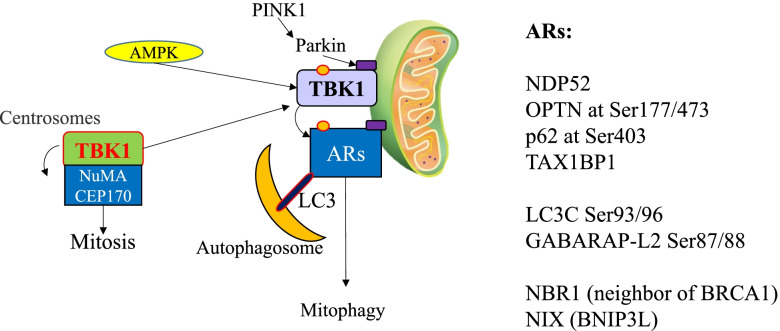
TBK1 regulates selective autophagy and xenophagy. TBK1 is activated during autophagy and xenophagy. Activated TBK1 promotes selective autophagy and xenophagy by phosphorylating some of the autophagic modifiers and receptors. This drives cargoes into autophosomes via interactions with both the cargoes and LC3. Interestingly, TBK1 accumulation on damaged cargo suppresses mitosis due to reduced TBK1 interactions with the key regulators of mitosis, NuMA and CEP170

Autophagy can be classified as either nonselective/bulk or selective types. Nonselective autophagy is normally induced by starvation, which degrades cellular substrates indiscriminately to refill the nutrients [191, 194, 195]. Selective autophagic processes include mitophagy, ER-phagy, pexophagy, aggrephagy and xenophagy, which mediate the degradation of damaged mitochondria, endoplasmic reticula, peroxisomes, protein aggregates, and intracellular pathogens, respectively [195, 196]. Mitophagy, ER-phagy and aggrephagy are critical for controlling the quality of mitochondria/metabolism, ER/protein synthesis and protein foldin g[197–199], whereas xenophagy is a process by which intracellular pathogens are eliminated [190]. During selective autophagy, Ubd cargoes are recognized by autophagic receptors including p62/SQSTM1, OPTN, NDP52/CALCOCO2, TAX1BP1, NBR1 (neighbor of BRCA-1), and NIX (BNIP3L). These receptors contain an LC3-interacting region (LIR) and an Ub-binding domain (UBZ), which facilitate autophagosome fusion through LC3-LIR binding and selective binding to Ubd cargo via the UBZ (Fig. 4).

During autophagy, TBK1 is activated by AMPK-mediated phosphorylation [200]. In addition, Ub-marked cargoes recruit TBK1 to sites of damaged cargoes or pathogens and induce TBK1 activation locally by autophosphorylation on Ser172 [7, 55, 201–203]. Phosphatidylserine-specific phospholipase PLA1A mediates full activation of TBK1 through recruitment to mitochondria and interaction with MAVS [89, 204]. TBK1 regulates autophagy by phosphorylating autophagic modifiers and receptors, thereby increasing their binding affinities. For example, TBK1 phosphorylates the autophagic receptors OPTN, NDP52, TAX1BP1 and p62 on their Ub-binding and LIR domains to facilitate the binding of autophagic receptors to Ubd cargoes and LC3 [3, 11, 31, 57, 77, 138, 167, 190, 205–208]. Phosphorylation of OPTN by TBK1 enhances its binding to Ub chains and promotes selective autophagy of damaged mitochondria [54]. TBK1 phosphorylates syntaxin 17 on Ser202 to control the initiation of autophagy [73]. In addition, TBK1 phosphorylates RAB7A on Ser72, which promotes the recruitment of ATG9 vesicles to damaged mitochondria [54]. TBK1 also phosphorylates LC3C on Ser93/96 and GABARAP-L2 on Ser87/88, preventing premature cleavage of LC3s from nascent autophagosomes by ATG4 [71]. Thus, TBK1 plays a critical role in the maintenance of normal mitochondrial metabolism by promoting mitophagy of damaged mitochondria [3, 22].

During viral infection, TRIM23 mediates TBK1-dimerization and activation, which is required for early autophagic induction and p62 phosphorylation for viral clearance [34, 56]. In response to an invasive bacterial infection, TBK1 phosphorylates NDP52 and contributes to autophagic maturation and the elimination of mycobacteria [4]. Furthermore, TBK1 regulates the integrity of pathogen-containing vacuoles and prevents bacterial egress into the cytosol, where hyperproliferation can occur [209]. Therefore, TBK1 is required for combating pathogens by linking inflammation/cytokine production and xenophagy. Because several adaptors and substrates of the TBK1 pathway such as STING and IRF3 are degraded during autophagy [5, 210], the TBK1-driven autophagic feedback loop is also responsible for the attenuation of cGAS-STING-IRF3-IFN signaling [5, 210]. TBK1 mediates crosstalk between energy sensing and inflammatory signaling pathways [39].

#### TBK1 is a critical mediator of cell proliferation

TBK1 promotes cellular survival and proliferation through activation of the AKT-mTOR, STAT3, and NF-κB signaling pathways. TBK1 also regulates mitosis by phosphorylating key regulators, including PLK1, CEP170, NuMA, Cdc20, and Cdh1a [80, 142, 163]. Evidence suggests that TBK1 is activated during mitosis and is necessary for cell division [6]. In proliferating cells, phosphorylation of TBK1 on Ser172 coincides with phosphorylation of histone H3, a mitotic indicator, and the kinetics of TBK1 activation are correlated to PLK1 phosphorylation on Thr210; PLK1 is an enzyme that regulates chromosomal segregation, spindle assembly checkpoint, and maintenance of genomic integrity [142, 211]. Hyper-activation of PLK1 causes excessive phosphorylation of BUBR1, CEP192, and INCENP, thereby resulting in mitotic dysregulation. Ser172 phosphorylation on TBK1 leads to TBK1 localization to centrosomes and mitotic spindles, where it regulates microtubule dynamics and mitosis. TBK1 phosphorylates the centrosome-associated CEP170 on multiple sites, resulting in the binding of CEP170 to the Kif2b microtubule depolymerase [80]. TBK1 also phosphorylates the NuMA mitotic apparatus protein on multiple sites, which is necessary for the latter to bind the microtubule motor dynein.

Cdc20 and Cdh1 are cofactors for the anaphase-promoting complex/cyclosome (APC/C) which regulates the exit from metaphase, segregation of sister chromatids and entry into anaphase [212]. In lung and breast cancer cell lines, TBK1 phosphorylates Cdc20 on Ser134 and Cdh1 on Thr20, Ser39, Ser42, Ser58, Ser131, and Ser151 [163]. Inhibition of TBK1 leads to centrosome amplification, mitotic defects, growth arrest, and apoptosis. Interestingly, OPTN and the PINK1/Parkin pathway recruit TBK1 to mitochondria during mitophagy, which results in a G2/M block due to the loss of TBK1 on centrosomes and mitotic spindles. Thus, TBK1 provides an intriguing connection between innate immune signaling, autophagy/mitophagy, and the cell cycle [6, 213].

### Studies of *Tbk1*-knockout mice

Germline *Tbk1-*knockout (*Tbk1*^−/−^) mice of a C57BL/6 background die of liver failure [161], whereas *Tbk1*^−/−^ mice of 129S5 background (*Tbk1*^Δ/Δ^) are viable but highly sensitive to LPS-induced inflammation and display neurodegenerative phenotypes [24, 187] . Tissue-specific *Tbk1* knockout mice have provided useful models to study the roles of Tbk1 in somatic cells. So far, tissue-specific deletion of *Tbk1* has been studied in several types of immune cells, including CD4^+^ T cells [2], CD11c^+^ dendritic cells (DCs), CD19^+^ B cells, and lysosome 2/M (*Lyz2*)-expressing myeloid cells [214], as well as villin-expressing intestinal epithelial cells, albumin-expressing hepatocytes, nestin-expressing neurons, and adiponectin-expressing adipocytes (Table 2).

**Table 2:** Summary of *Tbk1*-deficient mouse phenotypes

*Genotype*	*Knockout strategy*	*Phenotype*
Germline *Tbk1*^−/−^ [161]	Homologous-recombination targeting/knockout of exons 1-2 of *Tbk1*	Embryonic-lethal at E14.5 due to liver failure, resulting from aberrant death of hepatocytes induced by TNFα-RIPK1signaling.
Germline *Tbk1*^Δ/Δ^ [24, 187]	*Prm1*-Cre-mediated excision of *Tbk1* exon 2 to yield global truncation of Tbk1, mice of 129S5 background (lack of the *Tnfrsf1b* gene) to circumvent embryonic-lethal phenotype of *Tbk1* deficiency [187]	Exhibit mononuclear and granulomatous cell infiltrates in multiple organs and inflammatory cell infiltrates in their skin; hypersensitive to LPS stimulation [187]. Reduced weight gain and pancreatic abnormalities amid high-fat diet; increased insulin sensitivity due to absence of Tbk1-mediated inhibition of insulin receptor signaling [24].
*Tbk1*^*fl/fl*^*,* neuron-specific [207]	*Nestin*-Cre	ALS/FTD symptoms as demonstrated by cognitive and locomotor deficits. Resulting from impaired autophagy in motor neuron-like cells
*Tbk1*^*fl/fl*^, adipocyte-specific [39]	*Adiponectin*-Cre	Attenuates HFD-induced obesity by increasing energy expenditure, as TBK1 directly inhibits AMPK to suppress respiration and increase energy storage; increased inflammation and decreased insulin sensitivity because TBK1 represses NF-κB activity.
*Tbk1*^*fl/fl*^, myeloid cell-specific [215–218]	*Lyz2*-Cre	In IAV infection model, knockout mice display dampened immune response to IAV and improved survival. This is due to reduced recruitment of CD64^+^SiglecF^−^Ly6C^hi^ inflammatory macrophages, and reduced expression of inflammatory cytokines in the bronchoalveolar lavage fluid, and reduced expression of both IRF- and NF-κB-target genes in the lung [217]. In ALS model, knockout mice develop accelerated ALS due to the increased inflammatory cell infiltration, which induces motor deficits and axonal damage [216]. Eight month-old mice displayed adipocytic hypertrophy, increased M1/decreased M2 macrophage infiltration, and increased pro-IL-1β protein level in eWAT. Four week-old mice fed HFD developed liver abnormalities consistent with NAISH. Mice also displayed insulin-glucose axis dysfunction and increased disease severity in a DSS-induced colitis model [215].
*Tbk1*^*fl/fl*^, T cell-specific [2]	*Cd4*-Cre	Impaired T-cell migration (T_eff_ egress from draining lymph nodes) due to the enhanced activation of AKT-mTORC1 signaling axis. In a neuroinflammatory autoimmunity model, Tbk1 knockout in T cells represses the development of experimental autoimmune encephalomyelitis (EAE).
*Tbk1*^*fl/fl*^, dendritic cell-specific [154]	*CD11c*-Cre	Upregulation of costimulatory molecules, increased T-cell-priming activity and upregulation of a subset of genes by IFNAR. Mice develop autoimmune symptoms and display enhanced antitumor immunity.
*Tbk1*^*fl/fl*^, B cell-specific [78]	*Cd19*-Cre	Uncontrolled production of IgA and the development of IgA nephropathy-like disease; activation of the non-canonical NF-κB pathway in B cells.
*Tbk1*^*fl/fl*^, intestinal epithelia-specific [219]	*Villin*-Cre	Increased MT1 expression; increased number of Th17 cells in lamina propria; increased production of IL-1β by intestinal macrophages; increased number/size of intestinal neoplasms.
*Tbk11*^*fl/fl*^, hepatocyte-specific [144]	*Albumin*-Cre	Increased liver lipid due to reduced β-oxidation of acyl-CoAs/fatty acids; fasting-state mitochondrial localization of ACSL1 is impeded.

In CD4^+^ T cells, Tbk1 acts as a negative regulator of Akt signaling; Tbk1 phosphorylates Akt on Ser378, thereby inducing degradation of Akt. Loss of Tbk1 perpetuates aberrant activation of the AKT-Foxo1/AKT-mTORC1-S6K1 pathways, resulting in both enhanced T-cell activation and impaired T-cell migration [2]. T cell-specific deletion of *Tbk1* leads to increased sensitivity of T cells to activating stimuli, an increased fraction of the T-cell population being in either the T effector (T_eff_) or T memory (T_mem_) state at the expense of the T naïve (T_n_) population, and impaired exit of T_eff_ from lymph nodes [2].

Tbk1 in myeloid cells primarily restricts inflammatory responses. Myeloid-specific *Tbk1* knockout mice (*My-Tbk1*^*−/−*^) spontaneously developed adipose hypertrophy and metabolic disorders in old age due to the increased M1 macrophage infiltration and proinflammatory cytokine production (such as IL-6, TNF-α, and IL-1) in adipose tissue. Such mice are hypersensitive to high-fat diet (HFD)-induced hepatic inflammation, insulin resistance and non-alcohol-induced steatohepatitis (NAISH)-like fatty liver disease, as well as to dextran sulfate sodium (DSS)-induced experimental colitis. Mechanistic studies suggest that Tbk1 negatively regulates TLR-stimulated MAPK and IKK/NF-κB signaling and proinflammatory cytokine production. The disease phenotypes observed in *My-Tbk1*^*−/−*^ mice can be largely prevented by either genetic or pharmacologic inhibition of IL1 through IL-1R signaling, suggesting a critical role for Tbk1 in restricting TLR-stimulated inflammatory reactivation [215]. Another study reported that *My-Tbk1*^*−/−*^ mice developed an abnormal inflammation of the small intestine in old age characterized by mucosal infiltration of inflammatory macrophages [216]. In an ALS model with mutant human SOD1^G93A^ overexpression, *My-Tbk1*^*−/−*^ mice developed an accelerated ALS-like phenotype compared to SOD1^G93A^ *Tbk1*^+/+^ mice. This phenotypic expression consists of axonal destruction complicated by reduced integrity of myelin resulting from increased inflammatory cell infiltration [216]. However, in IAV infection model, *My-Tbk1*^*−/−*^ mice experience both decreased inflammation and mortality in response to viral infection when compared with wild-type mice, despite no differences in viral load. Compared to wild-type mice, *My-Tbk1*^*−/−*^ mice had fewer CD64^+^SiglecF^−^Ly6C^hi^ inflammatory macrophages, as well as reduced expression of both IFR3 and NF-κB target genes in the lung tissue, suggesting that Tbk1 is required for IAV infection-induced recruitment of CD64^+^SiglecF^−^Ly6C^hi^ inflammatory macrophages to the alveoli of the lung and inflammatory cytokine production [217]. These studies altogether point to context-specific roles for Tbk1 in different types of macrophages.

In CD19^+^ B cells, Tbk1 negatively regulates the non-canonical NF-κB pathway by phosphorylating NIK on Ser862, thereby triggering NIK degradation [78]. Mice with B cell-specific *Tbk1* deletion displayed an uncontrolled production of IgA and the development of human IgA nephropathy-like disease because Tbk1-NIK signaling modulates IgA class switching [78].

In CD11c^+^ DCs, Tbk1 suppresses the expression of IFNα-regulated genes and immunostimulatory molecules [154]. Mice with a DC-specific deletion of *Tbk1* displayed more T_mem_ cells and IFNγ^+^ T cells with reduced T_n_ cells in their spleens, suggesting Tbk1 in DCs restrains T-cell activation. Consistent with this notion, such mice developed autoimmune symptoms as they aged, yet displayed a remarkable ability to resist the neoplastic growth of xenografted melanoma, thymoma, and lymphoma [154]. The autoimmune and enhanced antitumor phenotypes seen in mice are associated with the elevated ratio of splenic T_mem/_T_n_ population.

In hepatocytes, Tbk1 mediates β-oxidation of acyl-CoAs/fatty acids via its interaction with ACSL1. Interestingly, this Tbk1-ACSL1 interaction only occurs when Tbk1 is inactive [144]. In the fasted state, Tbk1 complexes with ACSL1 in mitochondria to mediate β-oxidation; however, in the fed state, Tbk1 does not associate with ACSL1, allowing ACSL1 to translocate to the ER where it mediates the re-esterification and consequent storage of fatty acids. As such, hepatocyte-specific deletion of *Tbk1* in mice results in fatty liver disease due to a lack of TBK1-mediated sequestration of ACSL1 in mitochondria, thereby causing a defect in the β-oxidation of fatty acids.

In motor neurons, Tbk1 plays a critical role in the regulation of autophagy and, thus, of neuronal cell health [20]. Duan et al. demonstrated a mouse neuron-specific deletion of *Tbk1* results in the accumulation of intracellular p62^+^ protein aggregates and neurofibrillary/tau tangles in neurons, such that the affected mice display ALS/FTD-like symptoms [207].

In adipocytes, Tbk1 controls energy metabolism and suppresses inflammation. Tbk1 expression is upregulated in adipocytes of HFD-fed mice, which suppresses AMPK-mediated lipid oxidation/mitochondrial biogenesis and NIK-mediated non-canonical NF-κB pathway. Mice with adipocyte-specific deletion of *Tbk1* developed a T2D-like phenotype as demonstrated by increased insulin resistance/glucose intolerance and inflammation/macrophage infiltration of adipose tissues due to the activation of non-canonical NF-κB signaling. However, such mice are relatively resistant to HFD-induced obesity due to the elevated AMPK-mediated lipid oxidation and mitochondrial biogenesis [39, 220]. Interestingly, mice with whole body *Tbk1* knockout maintain insulin sensitivity because Tbk1 is a negative regulator of the IR [24]. Such mice are protected from HFD-induced weight gain, inflammation and diabetes. The distinct phenotype of adipocyte-specific *Tbk1* knockout mice and whole body *Tbk1* knockout mice suggests a critical role of Tbk1 in other cell types such as macrophages and that it might play an essential role in the regulation of insulin sensitivity and glucose tolerance.

### Role of TBK1 in the pathogenesis of cancer

Emerging evidence suggests that TBK1 plays critical yet tissue-variable roles in the pathogenesis of cancer [23, 221]. While *TBK1* mutations are not commonly reported in human cancers, increased TBK1 expression and/or aberrant TBK1 activity are reported in non-small cell lung cancer (NSCLC), pancreatic ductal adenocarcinoma (PDA), cholangiocarcinoma, clear cell renal cell carcinoma (ccRCC), adult T-cell leukemia, melanoma, esophageal cancer, and breast cancer, among others [142, 221–223]. In these cancers, TBK1 activity is positively correlated with disease progression thus serving as an indicator of poor prognosis, specifically in those tumors harboring activated K-RAS/N-RAS, suggesting TBK1 functions as an oncoprotein at least in these cancer types [16, 79, 142, 165, 174, 224–226]. The tumorigenic activity of TBK1 has been verified in several cancer models, suggesting that TBK1 may be an attractive molecular target for antineoplastic drugs [17, 81, 100, 225]. TBK1 can promote cancer development and progression via several mechanisms, including: 1) stimulating both survival and proliferation signals in cancer cells (i.e. cell-autonomous mechanism); 2) mediating the production of tumorigenic, immunosuppressive cytokines (i.e. autocrine-like mechanism); and 3) suppressing anticancer functions of the immune system by both upregulating the expression of immune checkpoint ligands (e.g. PD-L1) and perpetuating the inflammatory, macrophage-laden tumor microenvironment (i.e. cell-nonautonomous mechanism) [9, 51, 100, 222]. In addition, TBK1 in immune cells (e.g. DCs and CD8^+^ T cells) might antagonize antitumor immunity, thus promoting tumor development [154]. However, TBK1 is downregulated in uterine corpus endometrial carcinoma and oligodendroglioma, where it may function as a tumor suppressor. Therefore, carefully studying the roles of TBK1 in different types of cancer will help to develop a rationale for targeting TBK1 as part of an anti-neoplastic therapeutic strategy.

#### Molecular mechanisms by which TBK1 is upregulated and activated in cancer tissues

As is the case in normal cells, TBK1 activation in cancer cells can be stimulated by PAMPs, DAMPs, and inflammatory cytokines. In addition, some oncogenic kinases and receptor tyrosine kinases (such as K-RAS/N-RAS and AXL) can also activate TBK1 in neoplastic cells [23].

*KRAS* is mutated in a wide array of human cancers, including PDA, colorectal, NSCLC, endometrial cancers, and cholangiocarcinoma, most of which are aggressive and resistant to conventional antineoplastic therapies [227, 228]. TBK1 is susceptible to hyperactivation in those cancer cells harboring *KRAS*-activating mutations. RNAi screening identified TBK1 as a protein whose function is required for the survival of cancer cells which harbor activated K-RAS, implicating TBK1 and activated K-RAS as synthetic-lethal partners (i.e. *KRAS*-activating mutations are expected to sensitize the cells harboring them to TBK1 inhibition) [16]. Several potential mechanisms have been hypothesized regarding K-RAS-mediated activation of TBK1. For example, in many human cancer cell lines and mouse embryonic fibroblasts, activated K-RAS triggers TBK1 autophosphorylation on Ser172 by recruiting TBK1 to the exocyst where TBK1 appears to complex with Sec5 and RALB GTPases [229, 230]. In lung epithelial cells, activated K-RAS activates TBK1 via the induction of a complex containing TBK1, TBKBP1, CARD10, and PKCθ. PKCθ phosphorylates TBK1 on Ser710 in a TBKBP1- and CARD10-dependent manner [100]. In K-RAS-activated PDA, the AXL receptor tyrosine kinase induces TBK1 activity via Ras-RalB signaling [17].

#### TBK1 promotes the proliferation and survival of cancer cells by activating intrinsic signaling within cancer cells

**Fig. 5:**
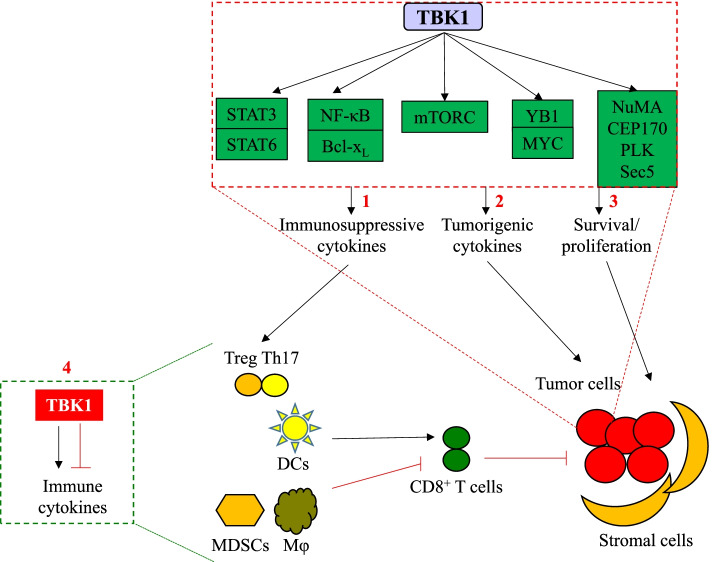
The role of TBK1 in the pathogenesis of cancer. In cancer cells, TBK1 promotes tumor cell growth by: 1) stimulating proliferation and survival signaling; 2) inducing the production tumor-promoting cytokines; and 3) promoting the secretion of immunosuppressive inflammatory cytokines. In immune cells, 4) TBK1 regulates the production of immunoregulatory factors that either inhibit or promote the anticancer effects of CD8^+^ T cells. However, the role of TBK1 in the antitumor activity of CD8^+^ T cells has not been adequately studied

In the cytoplasm of cancer cells, TBK1 drives tumor development and progression by stimulating cell survival and proliferation pathways, including AKT-mTOR1 [165, 229, 230], NF-κB [41, 160, 161, 183], p62/autophagy [3], MYC [231], and JAK/STAT [72, 100]. In addition, TBK1 can also induce the production of tumorigenic cytokines, including IL-6, which can promote cell survival and proliferation in an autocrine fashion [1, 222, 232] (Fig. 5).

In cancer cells harboring a *KRAS*-activating mutation, TBK1 is observed to promote cancer cell survival and proliferation by activating both the NF-κB and mTOR1 pathways [16, 68, 165]. Activation of NF-κB promotes the expression of anti-apoptotic Bcl-X_L_ while the activation of mTOR1 induces protein-synthetic pathways, both of which contribute to tumorigenesis [23]. TBK1 activates NF-κB signaling by phosphorylating critical regulators of the NF-κB pathway, including IkBα, RelA, and IKKβ [1, 39, 70, 233]. In addition, TBK1 has been shown to complex with multiple AKT-mTORC1-regulatory elements, including AKT, Raptor, RagD, and S6K [165]. TBK1 can activate mTORC1 signaling directly (by phosphorylating mTOR on Ser2481), or indirectly (by phosphorylating AKT on Tyr308 and Ser473, or by mTORC2-mediated phosphorylation of AKT on Ser473). AKT activates mTORC1 signaling via the canonical pathway [79, 229]. In addition, TBK1 can directly promote S6K C-terminal Thr421/Ser424 phosphorylation [165]. It was known that *KRAS*/*NRAS*-activating mutations promote the activation of both MEK-ERK and AKT-mTOR signaling in tumor cells [16, 18, 223]. The TBK1-dependent mTOR signaling activation in K-RAS/N-RAS-activated tumor cells suggests that TBK1-mTOR and MEK-ERK constitute parallel survival and proliferative signaling pathways in such tumor cells [18, 223, 234]. However, despite the biological plausibility of combination TBK1/MEK inhibition as an anticancer therapy, resistance to this regimen develops rapidly in mouse models of *KRas*^*G12D*^*Lbk1*^*null*^ and *KRas*^*G12D*^*Trp53*^*null*^ lung cancers due to the epigenetic-mediated upregulation of IGF1 and YAP1 [235]. Addition of the BET inhibitor JQ1 can inhibit both IGF1 and YAP1 signals, thereby resensitizing these tumors to combination TBK1/MEK inhibition [235]. Combination inhibition of TBK1, MEK, and BET (using MMB, trametinib, and JQ1, respectively) results in prolonged antitumor activity in both models.

In K-RAS-activated PDA, TBK1 functions downstream of AXL and supports both growth and metastasis of PDA cells by apparently reprogramming epithelial cell fate. In the *KRas*^*LSL-G12D/+*^*Cdkn2a*^*Lox/Lox*^*Ptf1a*^*Cre/+*^ PDA mouse model, *Tbk1* deletion in the malignant cells resulted in reduced tumor load and reduced metastatic behavior, indicating that Tbk1 activity contributes directly to the aggressive properties of pancreatic cancers. *Tbk1*-null cancer cells are more differentiated and less invasive than *Tbk1*-intact cancer cells in mice [17]. In primary melanoma patient samples, TBK1 is hyperactive in a subtype of BRAF/MEK inhibitor-resistant tumor cells; this subtype of melanoma displays hyperactive TLR/innate immune system signaling. TBK1 activation in such tumor cells is primarily stimulated by TGFβ, which promotes the survival of tumor cells by activating AKT and YAP signaling pathways. TBK1 protects YAP/TAZ from proteasomal degradation. Consequently, such melanoma cells are vulnerable to treatment with a TBK1 inhibitor [115, 225, 236].

However, TBK1 inhibits mTOR signaling in bone marrow-disseminated prostate cancers (PCa) [10, 237]. This mTOR-related inhibitory role for TBK1 was also observed in other types of somatic cells and T-cells in mice [8, 168]. TBK1 plays such a role by phosphorylating Raptor on Ser877 probably in a subcellular localization-dependent fashion [10, 15]. Bone metastatic lesions are associated with a poor prognosis and are detected in approximately 70% of patients with breast cancer or PCa and 15-30% of patients with carcinomas of the lung, colon, stomach, bladder, uterus, rectum, thyroid, or kidney [238]. Using the PCa PC3 cell line and a transplantation mouse model, Taichman’s laboratory demonstrated that PC3 cells compete with hematopoietic stem cells (HSC) for bone marrow niche occupancy [239]. Within HSC niches, PCa cells are resistant to conventional treatments because they are maintained in a cell cycle-quiescent state (G_0_) and display a cancer stem cell-like phenotype. TBK1 expression in PCa cells is induced by stromal cell attachment, which inhibits mTOR-p70S6K signaling and contributes to their dormant/stem-like phenotype. *TBK1* knockdown induces the activation of mTOR-p70S6K signaling and increases the sensitivity of cancer cells in bone marrow niches to docetaxel treatment as demonstrated by a xenograft model [237].

In addition to NF-κB and mTOR, TBK1 can also promote cancer development and progression through other pathways. Loss of copy number of chromosome 3p is strongly correlated with the development of both inherited/familial and sporadic clear-cell renal cell carcinoma (ccRCC). The *VHL* gene, which encodes the pVHL tumor suppressor, resides on chromosome 3p and is frequently deleted in ccRCC tumors [240, 241]. As such, it is believed that loss of pVHL functionality is a critical driver of ccRCC. Hu et al. demonstrated that phosphorylation of TBK1, and thus TBK1 kinase activity, is increased in ccRCC tumor tissues and is required for the survival of *VHL*^null^ ccRCC cells [81]. Mechanistically, it was found that the EGLN1 prolyl hydroxylase mediates hydroxylation on Pro48 of TBK1, thereby enabling the pVHL-associated E3 Ub ligase machinery to complex with TBK1. Upon associating with the Pro48-hydroxylated TBK1, pVHL recruits PPM1B to dephosphorylate TBK1, thereby blocking the kinase activity of TBK1. In *VHL*^null^ ccRCC tumor cells, loss of *VHL* copy number results in hyperactivation of TBK1, which leads to increased autophagy, due to TBK1-mediated phosphorylation of p62/SQSTM1 on Ser366. Interestingly, *VHL*^null^ ccRCC cells rely on TBK1-p62-induced autophagy for their survival. As a consequence, TBK1 inhibition leads to synthetic lethality in *VHL*^null^ ccRCC cells [81].

In breast cancers, TBK1 expression was significantly higher in most breast tumor tissues compared to matched adjacent normal tissues as demonstrated by immunohistochemical staining of 171 breast cancer samples. Mechanistically, the N-terminal fragment of TBK1 (aa1-510) interacts with the DNA binding domain of estrogen receptor α (ERα) and phosphorylates it on Ser305, which consequently perpetuates the transcriptional activity of ERα. Increased expression of TBK1 is positively correlated with ERα and cyclin D1 expression, as well as phosphorylation of ERα on Ser305. TBK1 inhibition sensitizes breast cancer cells to tamoxifen-induced cell death [174].

In acute myeloid leukemia, higher levels of TBK1 and IKKε expression were detected in leukemic cells compared to CD34^+^ HSPCs isolated from healthy donors. Genetic ablation of either *TBK1* or *IKKε* induces apoptosis in AML cells that express high levels of MYC, suggesting that both kinases are required for the survival of MYC^high^ leukemic cells. Pharmacologic inhibition of TBK1/IKKε suppresses AML development and progression in a xenograft model as demonstrated by a reduction in overall leukemic burden [231]. Mechanistic studies suggested that TBK1 and IKKε phosphorylate YB1 on Ser102, therein promoting the YB1-mediated expression of MYC. Compared to normal HSPCs, AML cells appear to rely more heavily upon TBK1/IKKε; this TBK1/IKKε addiction plausibly provides a selective therapeutic opportunity for AML patients via pharmacologic inhibition of TBK1/IKKε [18, 231].

#### TBK1 promotes the proliferation and survival of cancer cells by promoting autocrine cytokine signaling

In diffuse large B-cell lymphoma (DLBCL), *TBK1* mRNA levels negatively correlate with prognosis in both germinal center and non-germinal center types of DLBCL [242]. In DLBCL cells, TBK1 mediates the activation of canonical NF-κB signaling via phosphorylation of p65/RelA on Ser536, which promotes the production of IL-10, CCL3, and CCL4; these cytokines promote tumorigenesis by stimulating STAT3 activation. As such, TBK1 is predicted to mediate the activation of NF-κB/STAT3 in DLBCL, thereby promoting disease progression. As demonstrated in cell line and patient sample studies, treatment with the TBK1/IKKϵ inhibitor DMX3433 attenuated canonical NF-κB signaling and decreased DLBCL cell viability [224]. Furthermore, TBK1 is required for the survival of HTLV-1 (Human T-Lymphotropic Virus type 1)-transformed T lymphocytes through the maintenance of STAT3 activity. Thus, inhibition of TBK1/IKKε induces apoptosis in HTLV-1^+^ T lymphoma cells [226]. The study of several human cancer cell lines by Korherr et al. revealed that TBK1-IRF3 signaling can be activated in response to hypoxia. TBK1 mediates the production of RANTES, IL-8, and other proangiogenic factors in these cells. These factors stimulate tumor angiogenesis by promoting the proliferation of endothelial cells, suggesting that TBK1 may function as an angiogenic effector [243].

#### TBK1 promotes tumor development through an immunosuppressive tumor environment

The tumorigenic activities of TBK1 are not only mediated by intrinsic mechanisms as described above, but also by attenuating the antitumor functions of the immune system, which occurs through upregulation of immune checkpoint ligands and the maintenance of an immunosuppressive molecular signature in the tumor microenvironment [100].

In several types of murine cancer models, TBK1 mediates the production of immunosuppressive cytokines and chemokines which induce the expression of the immune checkpoint ligand programmed cell death-ligand 1 (PD-L1) and/or the accumulation of immunosuppressive cells such as myeloid-derived suppressor cells (MDSCs). For example, in a mouse model with *KRas*-activated lung cancers, Tbk1 induces local immunosuppression by facilitating EGF-induced PD-L1 expression on tumor cells. Deletion of Tbk1 in tumor epithelial cells reduces the number of both PD-L1 expressing cells and MDSCs in the tumor microenvironment, the reduction being associated with a local increase in CD8^+^ T-cells [100]. In HPV^+^ cervical cancer cells, high interferon-inducible 16 (IFI-16) expression is associated with increased PD-L1. Using a xenograft model, Cai et al. demonstrated that IFI-16 promotes cervical cancer progression by upregulating PD-L1 in the tumor environment via the STING-TBK1-NF-κB pathway [51]. Similarly, in hepatocellular carcinoma (HCC) patients, TBK1 expression was found to be higher in tumor tissues compared to adjacent normal tissues [222]. High TBK1 expression in tumor tissues is associated with reduced tumor-infiltrating CD8^+^ T-cells and increased immunosuppressive markers; thus, upregulation of TBK1 serves as a negative prognostic indicator for HCC patients. Interestingly, the TBK1 antagonist GSK8612 inhibits HCC cell growth only in immunocompetent animal models due to its effects on CD8^+^ T-cell infiltration [222]. In the mouse model of cerulein-induced pancreatitis/K-Ras^G12D^ PDA, TBK1 in the tumor cells promotes neutrophil recruitment and T-cell infiltration by stimulating the production of cytokines, such as CCL5 and IL-6, and the upregulation of PD-L1 [9]. This was supported further by another study that demonstrated that the loss of *Tbk1* significantly enhanced the antineoplastic effect of PD-1 blockade in melanomas and in other models of malignancy [244].

#### The protumor and antitumor activities of TBK1 in immune cells

Within immune cells such as DCs, cytotoxic T-cells, and macrophages, the role of TBK1 signaling in the anti-pathogen immune-response has been well-documented; however, the role of TBK1 signaling in anticancer immunity has only be assessed in DCs [17, 245]. DC-specific *Tbk1* knockout mice developed autoimmune symptoms such as aberrant T-cell activation, splenomegaly, and lymphadenopathy, as well as lymphocytic tissue infiltrates. Using a B16-OVA melanoma cell implantation model, Xiao et al. demonstrated that such mice display an enhanced ability to resist neoplastic growth coupled with an increased responsiveness to PD-1/PD-L1 blockade [154]. Mechanistically, TBK1 activates STAT3 signaling in DCs, thereby attenuating IFNAR-STAT1 signaling and suppressing both the expression of costimulatory molecules and T cell-priming activity [154]. Nevertheless, the tumor immune roles of TBK1 in myeloid cells, T cells and B cells have not been evaluated in any cancer models [2, 78, 217]. Thus, the role(s) of TBK1 in immune cells with respect to the pathogenesis of cancer are largely unknown.

#### Tumor suppressive activity in APC-mutated intestinal tumors

*TBK1* mRNA has been observed to be increased in some human colorectal cancers. Such upregulation can, perhaps surprisingly, serve as a *favorable* prognostic indicator in rectal adenocarcinomas [219, 222]. These studies suggest that, in contrast to its tumorigenic role observed in most cancer types, TBK1 appears to possess tumor-*suppressive* activity in at least some colorectal cancers. Such anti-tumor activity of TBK1 has recently been discovered in a mouse model of *Apc*-mutated intestinal tumors [219]. *Apc*^Min/+^ develop intestinal polyps by 5 months of age. Yang et al. recently demonstrated that a villin^+^ intestinal epithelium-specific deletion of *Tbk1* enhanced intestinal tumorigenesis, as shown by significant increases in the number and size of intestinal polyps [219]. Further study suggested that Tbk1 in intestinal epithelial cells (IECs) suppresses NF-κB-mediated metallothionein 1 (MT1) production. Deletion of *Tbk1* in IECs leads to MT1 production within the lamina propria. MT1 stimulates macrophages to produce IL-1β, which then promotes the expansion of Th17 cells. Aberrant expanded Th17 cells promote tumor IEC growth by producing Th17 cytokines [219].

### TBK1 as a chemotherapeutic target for cancer treatment

Owing to the scope of its involvement and function in processes both within and outside the cancer cells, TBK1 has gained considerable interest as a possible drug target for cancer treatment. Many TBK1 inhibitors have been developed (Table 3), most of them being dual inhibitors of both TBK1 and IKKε. The antineoplastic effects of several TBK1 inhibitors have been assessed in vitro (Table 4) and evaluated in animal models (Table 5). Most of these TBK1 inhibitors have displayed anticancer activities in animal models and shown synergism with other anticancer therapies, including temozolomide (TMZ) and MEK/BET inhibition. Despite abundant in vivo data from pre-clinical investigations supporting the use of AMX as anticancer therapy, the only TBK1 inhibitor to enter clinical trials thus far has been MMB. This is largely due to the effect of MMB on IKKε, TBK1 and JAK1/2.

**Table 3:** Summary of current TBK1 inhibitors

Compounds	Targets	Reference	Clinical Trial
Amlexanox (AMX)	TBK1/IKKε	[40, 246–252]	NCT01842282; NCT01975935
AZ13102909 (AZ909)	TBK1	[223]	
BAY-985	TBK1/IKKε	[253]	
BX795	TBK1/IKKε Aurora B NUAK1 PDK1	[254–256]	
Compound I	TBK1/IKKε	[244]	
Compound II	TBK1/IKKε	[229, 257]	
DMX-14	TBK1/IKKε	[258]	
GSK8612	TBK1	[259]	
MDK10496	TBK1/IKKε	[260]	
MMB/CYT387/GS-0387	TBK1/IKKε ACVR1 ALK2 JAK1/2	[231, 261–267]	NCT02101021; NCT02206763; NCT02258607; NCT04173494/MOMENTUM; NCT01969838/SIMPLIFY1; NCT02101268
MPI-0485520	TBK1/IKKε	[268]	
MRT67307	TBK1/IKKε ULK1/2	[99]	
MRT68601	TBK1	[255]	
UNC6587/Cereblon-TBK1 PROTAC	TBK1	[81]	
15a	TBK1/IKKε Aurora A GSK3β Aurora A PDK1	[269]	
200A	TBK1/IKKε	[270]	
3i/pVHL-TBK1 PROTAC	TBK1	[271]

**Table 4:** Summary of the in vitro antineoplastic activities of TBK1 inhibitors

Molecule	Findings	Reference
AMX	Inhibits growth of ALL cells (RS4;11 and SEM), displays synergism with TMZ in glioblastoma cells (U87MG) and patient samples, and attenuates the metastatic phenotype in prostate cancer cells (PC3 and DU145).	[247, 272, 273]
Compound II	Can induce apoptosis in lung cancer cells (HCC44, H1993, H2073, and H441), including H358 (K-RAS^G12C^) and SK-LU-1 (K-RAS^G12D^).	[229]
AZ909	Significantly attenuates the growth of N-RAS-activated melanoma cells (WM1366, SBcl2, and WM1346), displays synergism with MEK inhibitors.	[223]
MMB/CYT387/GS-0387	Decreases viability of AML cells (OCI-AML5, MOLM13, MOLM14, and KASUMI-1).	[15]
UNC6587 PROTAC	Selectively slows the growth of *VHL*^null^ ccRCC cells (UMRC6) while leaving VHL^+/+^-restored UMRC6 cells unaffected.	[80]

**Table 5:** Summary of the in vivo antineoplastic activities of TBK1 inhibitors

Compounds	Findings	References
AMX	Antitumor efficacy in mouse models of melanoma, glioblastoma, pro-B-cell leukemia, prostate cancer, and K-RAS-activated/CTLA4 blockade-resistant lung cancer. AMX and TMZ given in combination display synergism in human glioma cells in xenografts.	[100, 246, 249, 250, 252, 274]
Compound I	Antitumor capabilities when combined with PD-L1 blockade in mouse models of colorectal carcinoma.	[244, 270]
GSK8612	Inhibits HCC development in animal models by attenuating the production of immunosuppressive cytokines, consequently allowing enhanced infiltration of CD8^+^ T cells into the tumor.	[222]
MMB	Affords increased survival and decreased spleen size in mouse models of ovarian carcinoma and AML, respectively, and displays synergism with trametinib in suppressing PDA growth in animals.	[231, 275, 276]
200A	Anticancer activity in mouse models of squamous cell carcinoma.	[244, 270]

Oral AMX has been evaluated in the clinical setting, but only for the treatment of metabolic disorders, specifically NAFLD, obesity, and T2D (Table 3) [40, 262, 265, 266, 277, 278]. Oral administration of AMX in doses of 25-50 mg TID in a 12-week trial produced a statistically significant decrease in both serum HbA1c and fructosamine levels. However, these improvements in metabolic parameters seem to be lost upon discontinuation of the drug. Some patients in both trials displayed rashes diagnosed as perivascular inflammation, a pathology similar to the inflammatory skin lesions seen in *Tbk1*^∆/∆^ mice, suggesting an on-target side effect of TBK1 inhibition [187, 279]. While these studies demonstrated that oral AMX is safe, well-tolerated, and effective in ameliorating metabolic abnormalities, the efficacy of AMX as an antineoplastic agent is unknown but warrants clinical investigation [247, 280].

MMB has been evaluated for the treatment of metastatic PDA, EGFR- or K-RAS-activated NSCLC (Table 3) [18, 261–267]; however, oral MMB at doses of 100-300 mg QID did not show any clinically meaningful benefit, neither as monotherapy nor in combination with other drugs. The pharmacodynamics studies indicated that even with average maximal plasma concentrations of ~ 300 ng/mL, TBK1 was not inhibited to any therapeutically-significant extent [18, 262, 264–267]. One study with mouse RAW 264.7 gamma NO(−) macrophages suggested that MMB concentrations between 500 nM and 1 μM are required to block the kinase activity of TBK1 [18]. MMB has also been evaluated for the treatment of myelofibrosis, a disease associated with mutations and aberrant activation JAK2 signaling. In three phase 3 trials (two SIMPLIFY trials and one MOMENTUM trial), MMB displayed significant and promising treatment effects as demonstrated by reduced total symptom score and spleen volume as well as increased transfusion independence of patients for at least 12 weeks [263, 264, 281]. However, such treatment effects with MMB are primarily ascribable to the inhibition of JAK1/2 and ACVR1 signaling rather than inhibition of TBK1 signaling. It is also important to mention that MMB is metabolized differently across different mammalian groups, mainly undergoing morpholine ring oxidation in humans and amide hydrolysis in dogs/rats, thus yields different predominant metabolites between dogs/rats and humans [267]; the most abundant metabolite of MMB in humans, M21, is pharmacologically-active against JAK1/2 and ACVR1, whereas the most abundant MMB metabolite in dogs/rats, M19, is not. This finding is acknowledged to emphasize the reality that in vivo models, even dogs and large rodents, do not always translate directly to humans. As such, variations in drug metabolism between test and target species must be determined in any medicinal chemistry endeavor.

### Concluding remarks

Many diverse studies have demonstrated that TBK1 plays a role in not only innate immunity and metabolism, but also in cancer development and progression. In most cancer types, TBK1 functions oncogenically, serving to promote tumorigenesis through both cell-intrinsic and cell-extrinsic mechanisms. Thus, blocking the action of TBK1 in most cancer cells is expected to attenuate the invasive/malignant phenotype. However, owing to the critical role of TBK1 in normal immune physiology/activation, the inhibition of TBK1 could impair antitumor immunity. Interestingly, the genetic inhibition of *Tbk1* in DCs enhances antitumor immunity and arrests tumor development [154]. The roles of TBK1 in tumor immunity in other immune cells, including T and B cells as well as monocytes/macrophages, have not been sufficiently evaluated. While systemic inhibition of TBK1 augments antitumor immunity in most cancer types tested, particularly with concomitant immune checkpoint blockade, detailed analyses of the role of TBK1 in immune, tumor, and tumor microenvironment/stromal cells will be necessary to determine the advisability of targeting TBK1 as an antineoplastic therapeutic approach.

Cancer stem cells are unique types of cancer cell that are responsible for disease initiation, progression, and relapse [282, 283]. Compared to bulk tumor cells, cancer stem cells are relatively resistant to conventional therapies, likely due to their modulated metabolism. In examining its established involvement in mitophagy and mitochondrial metabolism, we hypothesize that TBK1 may contribute to the initiation and/or maintenance of the cancer stem cell phenotype [6, 54, 284]. As such, elevated TBK1 expression is associated with a poor prognosis in many cancer types. Future studies should evaluate the role of TBK1 in cancer stem cells and chemoresistance with the goal of determining whether pharmacologic inhibition of TBK1 can sensitize cancer stem cells to chemotherapy as a means of preventing disease relapse.

Much of the current literature suggests TBK1 inhibition could be an effective way to decrease cancer cell viability and invasiveness, across a remarkable range of cancer types, excluding those arising from villin^+^ intestinal epithelium [219]. Yet, it is still not entirely clear how cells/tissue types vary in their sensitivity to TBK1 inhibition. With respect to mutational status, some studies indicate that cancer cells harboring *KRAS*-activating mutations are vulnerable to K-RAS-TBK1 synthetic lethality, specifically when combined with ERK-MAPK inhibitors [9, 16, 18]. However, such effect is temporally due to BET-mediated drug-resistance. Thus, the addition of a BET inhibitor to the combination regimen of TBK1 and MEK inhibitors might prevent drug resistance and lead to prolonged antitumor activity. In addition, ccRCC cells harboring *VHL* deletions are inherently more sensitive to TBK1 inhibition, as the loss of VHL appears to cause cells to become addicted to TBK1 to perpetuate tumorigenic autophagy [60]. Detailed analyses of the roles of TBK1 with respect to both cancer type and mutational status will be required to best inform the investigation of TBK1 inhibitors in the treatment of cancer. Of clinical relevance, data from the villin^+^ intestinal epithelium-specific knockout of *Tbk1* suggest that the use of TBK1 inhibitors would be contraindicated in at least some patients with gastrointestinal carcinomas [219].

Finally, despite the significant antitumor effects of TBK1 inhibition demonstrated by in vitro culture systems and animal modeling, clinical benefit from the use of TBK1 inhibitors as cancer therapy was not observed with MMB and has yet to be investigated with AMX and others. Thus far, only MMB—an inhibitor of TBK1, IKKε, JAK1/2, and ACVR1/ALK2—has been evaluated in clinical trials. In multiple clinical trials, it was demonstrated that MMB failed to provide any appreciable benefit to metastatic patients, likely because it failed to adequately inhibit TBK1. Thus, the potency (e.g. IC_50_) and selectivity (e.g. TBK1*-*selective versus TBK1/IKKe dual) should be considered when choosing which TBK1 inhibitors to pursue in clinical trials for cancer therapy. As well, perhaps existing but ineffective TBK1 inhibitors (e.g. MMB) can be optimized with medicinal chemistry research before being disregarded entirely. Most TBK1 inhibitors also inhibit IKKε. Inhibition of both TBK1 and IKKε might provide better antineoplastic efficacy since both kinases have been implicated in oncogenesis. Given their high degree of sequence homology, inhibition of either TBK1 or IKKε alone may lead to compensatory hyperactivation of the untargeted congener, thereby circumventing the drug’s inhibitory effect. Although, as TBK1 and IKKε may be non-redundant in some cases, further evaluation is needed to determine if and when, specifically, TBK1- or IKKε-selective inhibitors would be preferred versus TBK1/IKKε dual inhibitors. It is clear that additional, more detailed studies are required to elucidate the distinct roles of both TBK1 and IKKε across cancer types and mutational statuses, specifically within immune cell physiology.

## Data Availability

Not applicable.

## Abbreviations

ACSL1: Acyl-CoA synthetase long-chain family member 1
AIM2: Absent in melanoma-2
ALRs: AIM2-like receptors
AMBRA1: Activating molecule in beclin-1-regulated autophagy
AMX: Amlexanox
ASD: Murine adipocyte (*Adiponectin*)-specific deletion of *Tbk1*
AZ909: AZ13102909
CCD: Coiled-coil domain
ccRCC: Clear-cell renal cell carcinoma
CDS: Cytosolic DNA sensors
DAI: DNA-dependent activator of IRFs, also known as DLM-1 or Z-DNA binding protein 1 (ZBP1)
DAMPs: Damage-associated molecular patterns
DSS: Dextran sulfate sodium
DUBs: deubiquitinating enzymes/deubiquitinases
eWAT: Epididymal white adipose tissue
IκB: Inhibitor of nuclear factor-κB
IAV: Influenza A virus
IFN: Interferon
IFI-16: Interferon-inducible-16
IFIT3: IFN-induced protein with tetratricopeptide repeats 3
IKK: IκB kinase
IR: Insulin receptor
HFD: High-fat diet
HSE: Childhood herpes simplex virus-1 encephalitis
HTLV-1: Human T-lymphotropic virus-type 1
IEI: Inborn error(s) of immunity
KD: Kinase domain
Lyz2: Lysozyme 2/Lysozyme M/LysM
LZ: Leucine zipper
MAVS: Mitochondrial antiviral signaling adaptor, also known as IPS-1, VISA, or Cardif
MDA-5: Melanoma differentiation-associated gene 5
MMB: Momelotinib, CYT387, GS-0387
MyD88: Myeloid Differentiation factor 88
NAFLD: Nonalcoholic fatty liver disease
NAISH: Non-alcohol-induced steatohepatitis
NAK: NF-κB-activating kinase (see also: TBK1)
NAP1: NAK-associated protein
NBR1: Neighbor of *BRCA1* gene 1
NDP52: Nuclear dot protein 52 kDa
NLRs: NOD-like receptors
NRBF2: Nuclear receptor-binding factor 2
NSCLC: Non-small cell lung cancer
OPTN: Optineurin
PAMPs: Pathogen-associated molecular patterns
PDA: Pancreatic ductal adenocarcinoma
PDE3B: Phosphodiesterase 3B
PIK3C3/VPS34: Phosphatidylinositol 3-kinase catalytic subunit type 3
PIK3R4/VPS15: Phosphoinositide 3 kinase regulatory subunit 4
PLK1: Polo-like kinase 1
Poly-Ub: Polyubiquitin
Poly-Ubn: Polyubiquitination
PRR: Pattern-recognition receptor
RIG-I: Retinoic acid-inducible gene I
RLRs: RIG-I-like receptors
SINTBAD: Similar to NAP1 TBK1 adaptor (synonymous with TBKBP1)
SSD: Scaffold dimerization domain
T2D: Type II diabetes
TAPE: TBK1-associated protein in endolysosomes
TANK: TRAF family member-associated NFKB activator (HGNC Symbol), also called TRAF- interacting protein (I-TRAF)
TAX1BP1: Tax1 binding protein 1
TBK1: TANK-binding kinase 1
TBKBP1: TBK1-binding protein 1 (synonymous with SINTBAD)
TLRs: Toll-like receptors
TMZ: Temozolomide
TRAF: TNF receptor-associated factor
TRIF: TIR-domain-containing adapter-inducing interferon-β, also known as TICAM1
TRIP: TRAF-interacting protein
Ub: Ubiquitin
Ubd: Ubiquitinated
Ubn: Ubiquitination
ULD: Ubiquitin-like domain
